# Multi-faceted attributes of salivary cell-free DNA as liquid biopsy biomarkers for gastric cancer detection

**DOI:** 10.1186/s40364-023-00524-2

**Published:** 2023-10-10

**Authors:** Neeti Swarup, Jordan Cheng, Irene Choi, You Jeong Heo, Misagh Kordi, Mohammad Aziz, Akanksha Arora, Feng Li, David Chia, Fang Wei, David Elashoff, Liying Zhang, Sung Kim, Yong Kim, David T.W. Wong

**Affiliations:** 1 School of Dentistry, University of California, Los Angeles, Los Angeles, CA 90095 USA; 2 The Samsung Advanced Institute for Health Sciences & Technology (SAIHST), Samsung Medical Center, Sungkyunkwan University School of Medicine, Seoul, 06355 Republic of Korea; 3 Indraprastha Institute of Information Technology (IIIT), Delhi, India; 4 Department of Pathology and Laboratory Medicine, David Geffen School of Medicine, University of California, Los Angeles, Los Angeles, CA 90095 USA; 5 Department of Medicine, Biostatistics and Computational Medicine, University of California Los Angeles, Los Angeles, CA 90095 USA; 6 Department of Surgery, Samsung Medical Center, Sungkyunkwan University School of Medicine, Seoul, 06355 South Korea

**Keywords:** Cell-free DNA, Salivary cell-free DNA, Liquid Biopsy, Fragmentomics

## Abstract

**Background:**

Recent advances in circulating cell-free DNA (cfDNA) analysis from biofluids have opened new avenues for liquid biopsy (LB). However, current cfDNA LB assays are limited by the availability of existing information on established genotypes associated with tumor tissues. Certain cancers present with a limited list of established mutated cfDNA biomarkers, and thus, nonmutated cfDNA characteristics along with alternative biofluids are needed to broaden the available cfDNA targets for cancer detection. Saliva is an intriguing and accessible biofluid that has yet to be fully explored for its clinical utility for cancer detection.

**Methods:**

In this report, we employed a low-coverage single stranded (ss) library NGS pipeline “Broad-Range cell-free DNA-Seq” (BRcfDNA-Seq) using saliva to comprehensively investigate the characteristics of salivary cfDNA (ScfDNA). The identification of cfDNA features has been made possible by applying novel cfDNA processing techniques that permit the incorporation of ultrashort, ss, and jagged DNA fragments. As a proof of concept using 10 gastric cancer (GC) and 10 noncancer samples, we examined whether ScfDNA characteristics, including fragmentomics, end motif profiles, microbial contribution, and human chromosomal mapping, could differentiate between these two groups.

**Results:**

Individual and integrative analysis of these ScfDNA features demonstrated significant differences between the two cohorts, suggesting that disease state may affect the ScfDNA population by altering nuclear cleavage or the profile of contributory organism cfDNA to total ScfDNA. We report that principal component analysis integration of several aspects of salivary cell-free DNA fragmentomic profiles, genomic element profiles, end-motif sequence patterns, and distinct oral microbiome populations can differentiate the two populations with a p value of < 0.0001 (PC1).

**Conclusion:**

These novel features of ScfDNA characteristics could be clinically useful for improving saliva-based LB detection and the eventual monitoring of local or systemic diseases.

**Supplementary Information:**

The online version contains supplementary material available at 10.1186/s40364-023-00524-2.

## Background

Saliva has demonstrated immense potential to be a viable biofluid for liquid biopsy (LB). [1] Saliva contains metabolic, [2] proteomic [3], and transcriptomic [4] components that are clinically useful for disease detection. Additionally, changes in oral health and dysbiosis in the oral cavity have been recorded in various diseases, especially gastroesophageal cancers (GC), [5–8] and these same changes can be reflected in the saliva. [9] Our group previously demonstrated discriminatory differences in extracellular RNA (exRNA) signatures in the saliva of GC patients. [4, 10] Additionally, aside from RNA and protein analysis, lung cancer pathognomonic circulating tumor DNA (ctDNA) is detectable in saliva. [11, 12] These findings suggest that saliva can be a useful biofluid for liquid biopsy.

Cell-free DNA (cfDNA) and ctDNA analysis in plasma has propelled liquid biopsy into a new phase for noninvasive disease detection. [13] However, the detection of ctDNA relies on assessing established genetic alterations identified from the genotypic analysis of cancer tissues. Developing a proper assay based on tumor tissue genotypes is technically difficult if specific cancers (such as GC) present with inter- and intrasample heterogeneity. Additionally, for general screening purposes, it can be challenging to determine the anatomic origin of the cancer based solely on the presence of ctDNA. One potential solution would be to develop multiple target assays to cover a substantial number of tumor mutations, but this would require significant technical advancement. More importantly, there could be biological reasons why cfDNAs harboring tumor tissue information are not coherently represented in biofluids and are not sufficiently present in all types and stages of cancers.

To address the limitations of ctDNA detection, researchers have investigated nonsomatic mutation-related patterns, such as methylation patterns, within circulating cfDNA fragments for disease detection. [14] Recently, the size distribution of cfDNA fragment length in plasma has demonstrated promising potential to differentiate cancer from noncancer patients. [13] Topological aspects of cfDNA have been described to be a function of nucleosomal positioning [15], the activity of nuclease enzymes [16] or the prevalence of potential G-quad complexes [17]. Nucleosomal positioning and nuclease activity contribute to the attributes of cfDNA, such as fragment lengths and [18] end motifs of DNA fragments [19]. These attributes show that nonsomatic mutation patterns have the potential utility to discern cancer and noncancer samples.

Nontargeted whole genome sequencing of cfDNA allows for the identification of these features in cfDNA fragments. Another advantage of nontargeted whole genome sequencing is that low coverage sequencing still allows for adequate profiling of these fragmentation metrics, lowering potential screening costs. However, the features of the cfDNA observed depend on processing methods such as extraction of DNA and processing procedures. [15, 20, 21] Multiple conformations of short and mononucleosomal length cfDNA have been observed in plasma, including single-stranded (ss), double-stranded (ds), jagged, etc., when different processing methods are used. We have recently described a unique NGS pipeline, Broad Range cell-free DNA-Seq (BRcfDNA-Seq), which permits extraction and processing of ultrashort ss cfDNA from plasma. [21] By application of BRcfDNA-seq, we show that saliva cfDNA (ScfDNA) is complex, similar to plasma cfDNA, in that it contains DNA of multiple conformations, such as ss, ds, jagged DNA, and nicked DNA. Additionally, as nonmutation attributes of plasma cfDNA have demonstrated clinical usefulness, [13, 19] we hypothesized that features of ScfDNA could be similarly valuable for differentiating between noncancer local and systemic diseases.

**Fig. 1:**
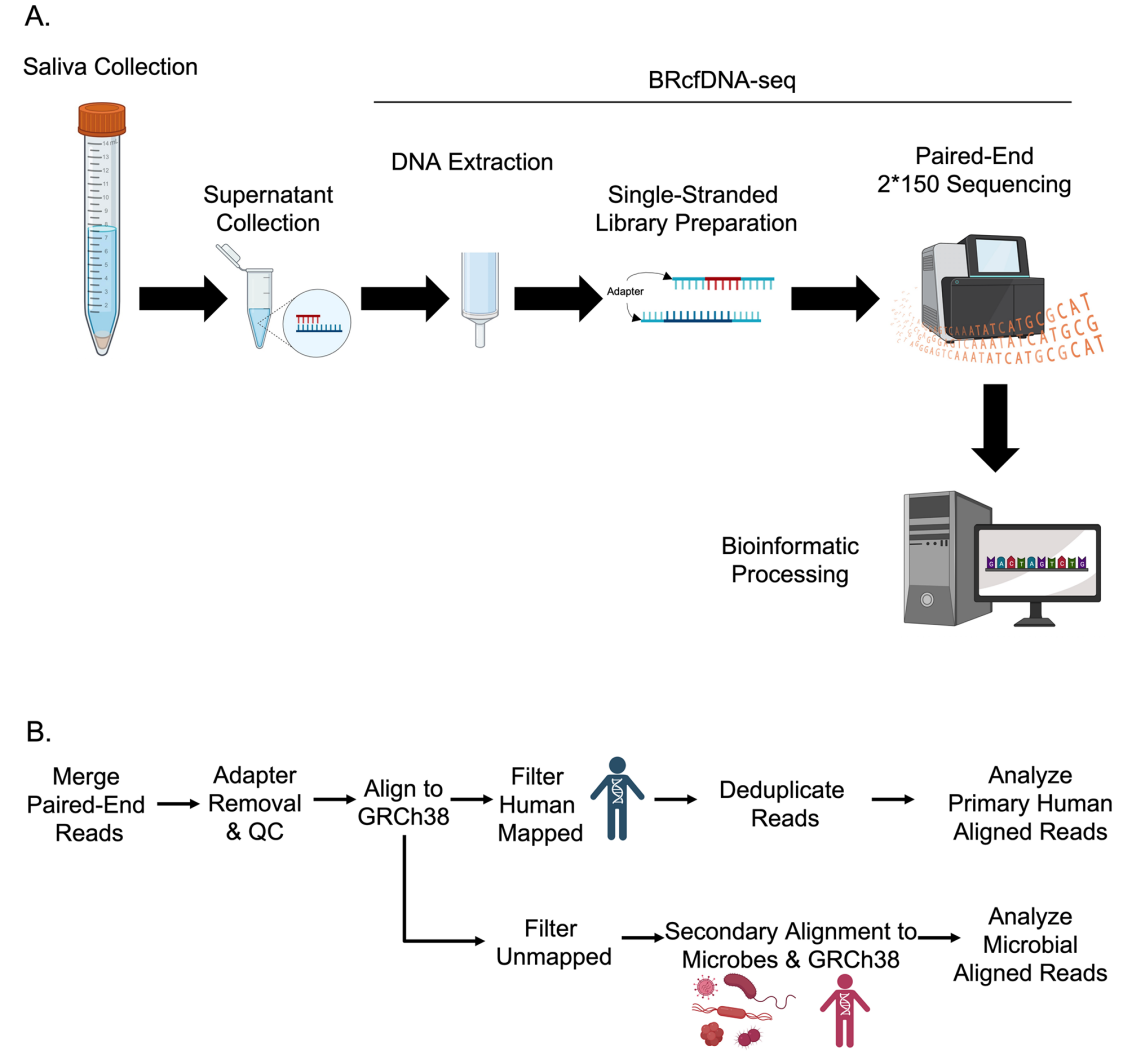
Workflow for processing and analysing Salivary Cell-Free DNA using BRcfDNA-seq (**A**) The BRcfDNA-seq laboratory workflow shows that after centrifugation, the saliva super supernatant is collected for extraction and using single-stranded library preparation, Illumina sequencing for Paired end reads 2*150 bp. (**B**) Overarching bioinformatic processing for ScfDNA.

As a proof-of-concept, we have tested the hypothesis that ScfDNA may have diagnostic utility as a biomarker by applying the size-agnostic extraction and ss NGS pipeline BRcfDNA-Seq (Fig. 1) to saliva samples from a cohort of 10 GC and 10 noncancer donors, and exploring the clinical utility of ScfDNA as a biomarker for GC could contribute to the development of new diagnostic tools.

## Methods

### Patients and extraction of cfDNA

For characterization of ScfDNA saliva sample from a healthy donor was collected using the standard operating procedure (SOP). [22] Fresh saliva was collected in a Falcon tube. The accumulated saliva was spun down at 2600 G for 15 min at 4 degrees Celsius. The supernatant was collected and transferred to a separate tube and taken for further processing to identify different ScfDNA conformations.

Saliva was collected from 10 diagnosed GC patients and 10 healthy volunteers. Samples were collected from Samsung Medical Center using the described SOP. Upon receiving the supernatant saliva, we again centrifuged it at 10,000xG for 15 min. Description of noncancer donors and cancer patients (Supplementary Tables 1–2).

### DNA extraction

DNA from 1 mL of saliva was extracted using the QIAmp Circulating Nucleic Acid Kit (Qiagen, 55,114) and Circulating microRNA protocol (QiaM). Proteinase-K digestion was carried out as instructed. Carrier RNA was not used. The ATL Lysis buffer (Qiagen, 19,076) was used as indicated in the microRNA protocol. The final elution volume was 20 µL (Fig. 1A).

### Nuclease digestions for analysis of strandedness

For characterization of ScfDNA, prior to library preparation, the cfDNA extracted from healthy donor was digested with various strand-specific nucleases. After the reaction, the DNA was purified by combining 30 µL of reaction buffer, 90 µL of SPRI-select beads, and 90 µL of 100% isopropanol and incubated for 10 min. The tube was placed on a magnetic rack for five minutes to allow the beads to migrate. The supernatant was discarded, and the beads were washed twice with 200 µL of 80% ethanol. Once the second ethanol wash was removed, the beads were left to air dry for 10 min. The beads were resuspended in 20 µL of Qiagen elution buffer (or 10 mM Tris-HCl pH 8).

#### ssDNA-specific digestion

Twenty microliters of cfDNA was combined with 3 µL of Exonuclease 1 (NEB, M0293S), 3 µL of 10x Exo 1 Buffer, and 4 µL of ddH2O, incubated for 30 min at 37 °C and heat inactivated for 15 min at 80 °C with 1 µL of 0.5 M EDTA.

#### dsDNA-specific digestion

Twenty microliters of cfDNA was combined with 2 µL of dsDNase (ArcticZyme, 70600-201) and 8 µL of ddH2O, incubated for 30 min at 37 °C and heat inactivated for 15 min at 65 °C with 1 mM DTT.

#### Nick repair analysis

Then, 20 µL cfDNA was combined with 1 µL PreCR Repair (NEB, M0309S), 5 µL ThermoPol Buffer (10x), 0.5 µL NAD+ (100x), 2 µL Takara 2.5 mM dNTP, and 21.5 ddH_2_O, incubated for 30 min at 37 °C and placed on ice.

### Single-stranded library preparation

ss DNA library preparation was performed using the SRSLYTM PicoPlus DNA NGS Library Preparation Base Kit with the SRSLY 12 UMI-UDI Primer Set and UMI Add-on Reagents and purified with Clarefy Purification Beads (Claret Bioscience, CBS-K250B-24, CBS-UM-24, CBS-UR-24, CBS-BD-24). Eighteen microliters of extracted cfDNA was used as input and heat-shocked as instructed. To retain a high proportion of small fragments, the low molecular weight retention protocol was followed for all bead clean-up steps. The index reaction PCR was run for 11 cycles (Fig. 1A).

### Double-stranded library preparation

For ds DNA libraries from healthy donor, NEB Ultra II (New England Bio, E7645S) was used with a 9 µL aliquot of extracted cfDNA according to the manufacturer’s instructions with some modifications: adapter ligation was performed using 2.5 µL of NEBNext® Multiplex Oligos for Illumina (Unique Dual Index UMI Adaptors RNA Set 1 – NEB, cat# E7416S); postadapter ligation purification was performed using 50 µL of purification beads and 50 µL of purification bead buffer, while the second (or post-PCR) purification was performed using 60 µL of purification beads (to retain smaller fragments). PCR was performed using MyTaq HS mix (Bioline, BIO-25,045) for 10 PCR cycles.

### Sequencing

Final library concentrations were measured using the Qubit Fluorometer (Thermo, Q33327), and quality was assessed using the Tapestation 4200 using D1000 High-Sensitivity Tapes (Agilent, G2991BA and 5067–5584). Final libraries were sequenced on an Illumina Novaseq 6000 instrument SP 300 (for the single healthy donor) or S1 (for GC cohort, cancer and noncancer donors) flow cell type (2 × 150 bp), yielding ~ 40 million reads per sample (Fig. 1A).

### Bioinformatic processing

Sequence reads were demultiplexed using SRSLYumi (SRSLYumi 0.4 version, Claret Bioscience), python package. Paired-end reads were merged with BBmerge (INFO). Fastq files were trimmed with (fastp, using adapter sequence AGATCGGAAGAGCACACGTCTGAACTCCAGTCA (r1) and AGATCGGAAGAGCGTCGTGTAGGGAAAGAGTGT (r2) and a Phred score of > 15. Then, sequenced reads were aligned against the combined human reference genome [GenBank:GCA_000001305.2] and LambdaPhage Genome [GeneBank:GCA_000840245.1] using Bowtie2 aligner. The unmapped sequences were filtered out and aligned to a microbial database using OneCodex. [23] The reads aligned to human reference were sorted and filtered using samtools (1.9 version). Reads were deduplicated by first moving the umi-tag using the bamtag tool from SRSLYumi (0.4 version), grouping with umi-tools (11.2 version), and removed using markduplicates from the Picard Toolkit (Quality control was performed with Qualimap (2.2.2c version). UMI-duplicate removal was performed first by moving the UMI-tag with srslyumi-bamtag (SRSLYumi), marking with umi-tools (11.2 version), and then removal with Picard (2.27.0 version). Functional peaks of human-aligned ScfDNA were called with macs2 (2.2.7.1 version) (Fig. 1B).

### Bioinformatic analysis

Human genome alignment files (.bam) were analysed using samtools, RIdeogram, and functional peaks HOMERannotatePeaks (version 4.11.1). Chromosomal binning was performed for chromosomal coverage and fragmentomic analysis, with each bin measuring 1 million bps. We used Flourish Studio to visualize genes contributing to ScfDNA using a chord diagram (https://flourish.studio). For the peak-valley index, we averaged the difference between the peak and the adjacent valley (on the right side of the peak) throughout the insert size histogram; peaks and valleys were identified using the peakdetect tool found in peakdetect GitHub (https://github.com/avhn/peakdetect). The lookahead value was set to 1, and the delta was set to 0.0001. The x-axis was set as the fragment length, and the y-axis was set to the calculated % reads. Based on the generated locations of peaks and valleys, we calculated the peak-valley index using the following equation:

$${\sum }_{i=1}^{n} \frac{{P}_{i}-{V}_{i}}{n}$$

where ${P}_{i}$ = peak at index $i$, ${V}_{i}$ = valley to the right of peak ${P}_{i}$ at index $i$, and $n$ = total number of peaks identified (Supplementary Fig. 2).

For microbial analysis, the unmapped reads, which were filtered out, were aligned to the microbial database hosted by OneCodex. OneCodex hosts whole shotgun metagenome assemblies of over 127k microbial species. In addition to microbial species, OneCodex has a human host reference for additional host alignment. Reads aligned to different taxa of microbes were identified based on abundance for different phylogenetic levels, and the reads aligned to the host genome were classified as second human-aligned reads. The second human-aligned reads were not considered for downstream analysis.

Additional analysis command lines, fragmentomics, end motif detection, and G-quad prevalence can be found in the BRcfDNA-seq Suite at WLab a GitHub, https://github.com/WlabUCLA/BRcfDNA-Seq. Raw sequencing data has been deposited at Sequence Read Archive, BioProject number PRJNA999038, and can be accessed from https://dataview.ncbi.nlm.nih.gov/object/PRJNA999038. Statistical analysis was performed using Prism8 (Version 8.4.0).

### Statistical analysis

For comparison between the two groups on a single parameter, such as peak-valley index, fragmentomic score, mitochondrial bulk, peaks per reads, Shannon entropy, G-Quad prevalence, microbial reads, and alpha diversity of microbes, we used Student’s t test with Welch’s correction. For fragmentomics, functional elements, and end motifs, we calculated significant regions of interest by performing multiple t tests with a false discovery rate of Q = 5 using the two-stage step-up method of Benjamini, Krieger, and Yekutieli. Volcano plots were generated, wherein each dot represents the q value and difference between the cancer and noncancer cohorts for each region of interest. Receiver operating curves and areas under the curve were plotted to identify the specificity and sensitivity of the differentiating abilities of individual features. Following the identification and discovery of significant features, multivariable analysis was performed using Clustvis. [24].

## Results

### Single- and double-stranded library preparation produces similar ScfDNA patterns

**Fig. 2:**
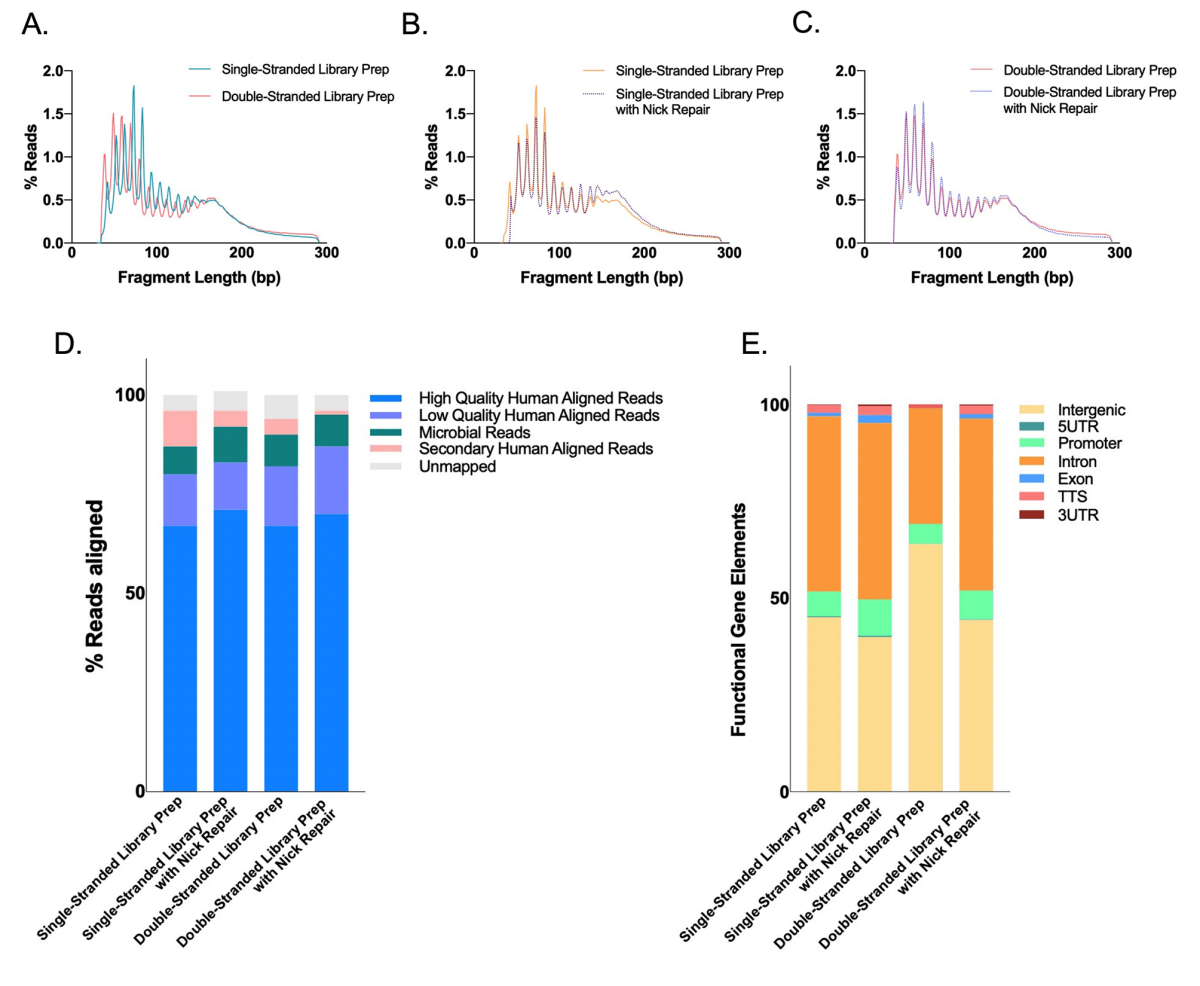
Characteristics of ScfDNA. ScfDNA insert size profile with multiple peaks and valleys using (**A**) Double-stranded library preparation (Peach solid line) and single-stranded library preparation (Turquoise solid line). (**B**) Single-stranded library preparation (Turquoise solid line) and Nick repair enzyme-treated ScfDNA (Turquoise dashed line). (**C**) Double-stranded library preparation (Peach solid line) and nick repair enzyme-treated ScfDNA (Peach dashed line). (**D**) Human and microbial origins of salivary cell-free DNA. Percentage of reads of ScfDNA mapping to humans and microbes. (**E**) Genomic element analysis using different library preparation methodologies is comparable to each other. Representative data obtained from single healthy donor

As single- and double-stranded library preparation inherently affects the incorporation of different types of DNA, we first examined whether it would affect the perceived characteristics of ScfDNA. Initially, ScfDNA was observed with a band at 100–200 bp on an electrophoresis gel following DNA extraction (Supplementary Fig. 1A). To clarify the conformation of ScfDNA, we used strand-specific DNA digestion, exonuclease for ss DNA, and Arcticzyme DsNase for ds DNA. We also repaired DNA fragments to identify whether the shorter fragments were derived from nicked DNA (dsDNA with breaks). Following enzymatic digestion and library preparation of extracted DNA from freshly collected saliva, we observed that ScfDNA demonstrated two bands at 300 and 200 bp (Supplementary Fig. 1B), suggesting the presence of fragments of varying lengths, i.e., mononucleosomal (~ 167 bp) and shorter cfDNA (~ 50–70 bp), since library adapters contribute ~ 150 bp. Subsequently, with nontargeted sequencing of ScfDNA libraries, we observed a peculiar, jagged profile of ScfDNA fragments with lengths ranging from 35 to 300 bp, regardless of library preparation methodology, suggesting the presence of ScfDNA fragments with lengths between the mononucleosomal length and short cfDNA length. (Fig. 2A-C). The majority of DNA obtained was below 200 bp in length. The peaks and valleys of the jagged pattern were observed at regular intervals of approximately 10 bp length within the 160 bp fragment length. A rightward shift of approximately 7 bp was observed in the ss library compared to the ds library (Fig. 2A). A similar rightward shift of ~ 3 bp has been observed in plasma with different library preparations (ss and ds). [18] This pattern may be attributed to the exposed DNA from the dyad structure of the DNA wound around the nucleosome. [18] Incorporating DNA repair for ScfDNA prior to ss or ds library preparation showed an increase in long cfDNA (> 100 bp) fragments, especially with ss library preparation (Fig. 2B&C). ScfDNA prepared using a ss library demonstrates a population of native nicked DNA with shorter DNA fragments, contributing to the shorter ScfDNA fragments. The results observed through different library preparations and enzymatic digestions suggest that different populations of cfDNA, ss, nicked (ds DNA with breaks), and jagged DNA exist in saliva. We then evaluated the possible origins of ScfDNA using human and microbial alignment. Approximately 70% of ScfDNA reads had a high-quality alignment to the human reference, while ~ 8% of reads aligned to the microbial reference for all library preparation methods, suggesting that ScfDNA maintains its identity despite library preparation methods (Fig. 2D). Similar to alignment, the human-aligned ScfDNA fragments retain their genomic identity; promoters, exons, introns, and intergenic regions (Fig. 2E) and genomic coordinates representing specific genes contributing to ScfDNA (Supplementary Fig. 1C) for different preparation methods.

To incorporate the heterogeneity of ScfDNA, for downstream analysis as a part of **BRcfDNA-Seq**, we decided to employ the ss library pipeline because it offered effective incorporation of all conformations (ss, ds, nicked, and jagged) without additional processing [25] within ScfDNA.

### Fragment size profile of ScfDNA differs between noncancer and GC patients

**Fig. 3:**
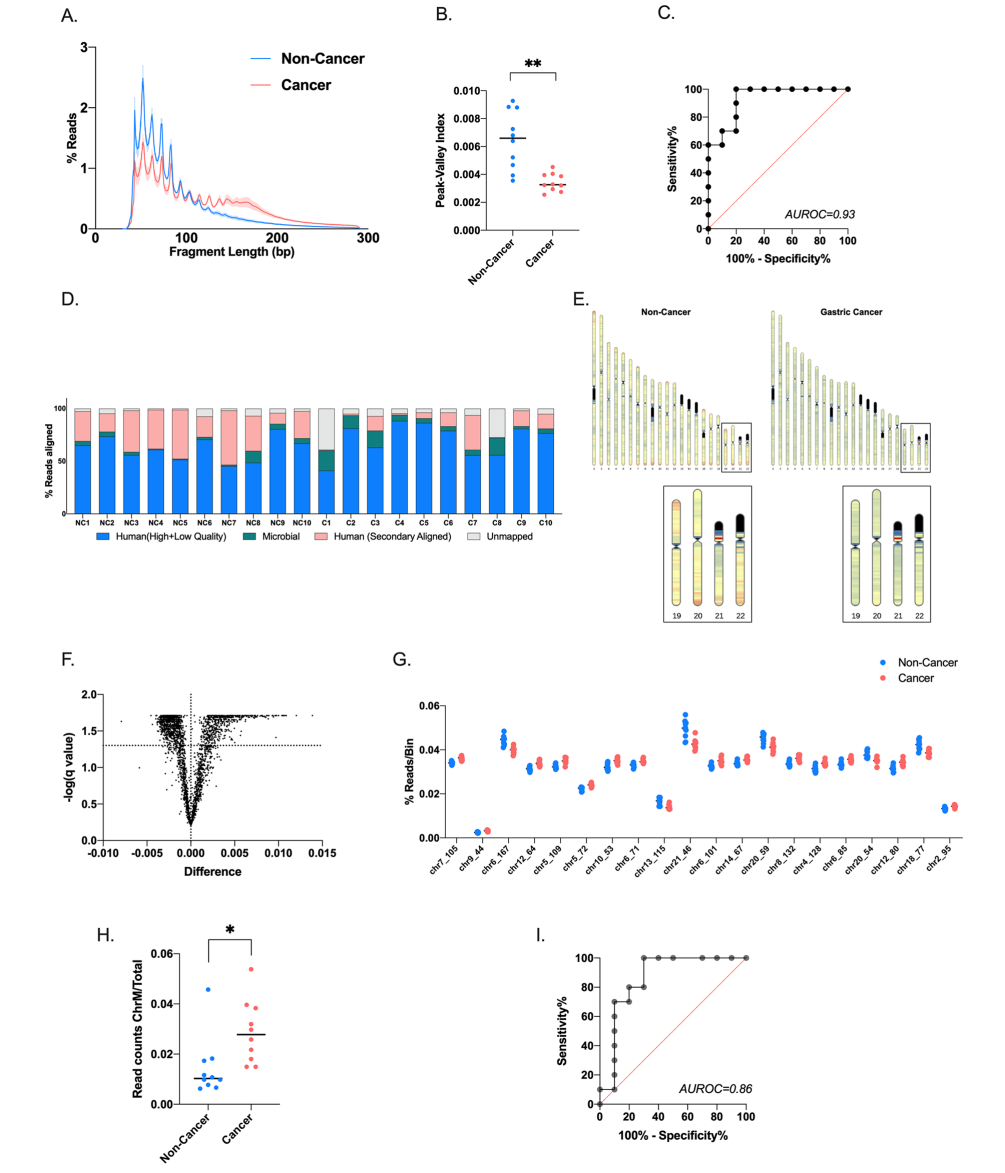
ScfDNA profile in cancer vs. noncancer donors. (**A**) ScfDNA insert size profile with multiple peaks and valleys below 100 bp using single-stranded library preparation for noncancer donors (Turquoise solid line) and an additional peak at ~ 167 bp for cancer donors (Peach solid line). (**B**) Peak-valley index of the ScfDNA fragment profile decreases in cancer subjects. The peak-valley index represents upward and downward inflections of the ScfDNA size distribution (Supplementary Fig. 2). P value = 0.0012, Student’s t test, Welch’s correction. (**C**) Receiver operating curve for prediction of gastric cancer based of peak-valley of insert size profile, area under receiver operating curve 0.93. (**D**) Human and microbial origins of salivary cell-free DNA from cancer and noncancer donors. Percentage of reads of ScfDNA mapping to humans and microbes. (**E**) Chromosomal locations of human mapped ScfDNA in noncancer and cancer donors increased ScfDNA reads at telomeric portions in noncancer donors when compared to cancer donors. Box shows zoomed in view of chr 19–22. (**F**) Volcano plot demonstrating over 1500 chromosomal locations significantly different between cancer and noncancer donors following multiple t tests, without considering the consistency of SD, false discovery rate Benjamini, Krieger and Yekutieli method, p value < 0.05, q value < 0.05. (**G**) Top 20 significant chromosomal locations, p value < 0.05, multiple t test, corrected FDR. (**H**) Differences in mitochondrial ScfDNA, t test p value < 0.014, (**I**) Area under receiver operating curve = 0.86

Due to the limitations of ctDNA mutation detection and the lack of driver somatic mutations in GC, we examined different aspects of cfDNA from saliva, such as fragment lengths and size distribution. Using BRcfDNA-Seq, we analysed the ScfDNA component from the supernatant of cell-free saliva from 10 noncancer and 10 GC subjects. A distinct mean fragment size profile ScfDNA was observed between noncancer and cancer samples (Fig. 3A). ScfDNA fragments less than 100 bp demonstrated multiple peaks, with consecutive peaks occurring at ~ 10 bp in noncancer and cancer samples. Additionally, ScfDNA derived from GC patients presented an additional plateaued peak at ~ 160 bps, which was missing from the noncancer donors. The peaks and valleys formed by the ScfDNA fragments demonstrated a peculiar jagged, peak-valley pattern (Fig. 3A). To quantify the peak-valley, we developed a “peak-valley index” to describe the average difference between the peak and valley (formula in methods) (Supplementary Fig. 2A). This index score was significantly higher in noncancer donors than in GC patients, with an AUROC of 0.93. (Fig. 3B&C). Interestingly, GC saliva was less jagged, contrasting the observation that urinary cfDNA from bladder cancer patients presented more peak-valley than that from noncancer donors. [26].

### Alignment patterns of GC ScfDNA are distinct from those of noncancer ScfDNA

Since human cells and microbiota are both present in saliva, we implemented a sequential alignment strategy. [20] After initial alignment to human reference, 61.85 **±** 11.54% of reads of noncancer samples and 70.80 **±** 15.84% of reads of cancer samples were aligned during the first run. The remaining unmapped/unaligned reads were then aligned to a microbial reference database (Fig. 3D). The total % of reads aligning to microbial references was lower in noncancer donors than in GC subjects, p value = 0.0361.

We examined the broad alignment behavior of ScfDNA fragments mapped to the human genome. For those with a human origin, ScfDNA fragments aligned throughout chromosomes 1–22 in both cancer and noncancer cohorts. Differences have been observed in the alignment of cfDNA to different portions of chromosomes depending on the disease status of the individuals. [27] In line with that observation, we found that more fragments of ScfDNA from noncancer donors align to the telomeric portions of the chromosomes and in the p and q arms of certain chromosomes. In contrast, ScfDNA fragments from cancer donors aligned more evenly throughout the chromosomes (Fig. 3E). We identified the portions of chromosomes with observable differences between the two groups. Following analysis of 2887 bins (1 million bps/bin), 1570 chromosomal bins demonstrated a significant difference between the two groups (Fig. 3F).

Human genomic reference has nuclear and mitochondrial components. Mitochondrial cfDNA has been reported to increase in cases of physiological stress, [28] trauma, and surgery, [29] and thus, we examined whether there were any noticeable changes in the reads that mapped to the mitochondrial genome. The histogram profile for human mitochondrial mapped sequences demonstrated a single peak contrasting with the two major and multiple minor peak profiles of ScfDNA. The mitochondrial cfDNA size distribution of GC subjects was shorter, with an average modal length of ~ 70 bp (Supplementary Fig. 2B). The % mitochondrial read contribution to ScfDNA was significantly higher in cancer than in noncancer subjects (Fig. 3H), p value = 0.014.

### Fragmentation pattern of ScfDNA

**Fig. 4:**
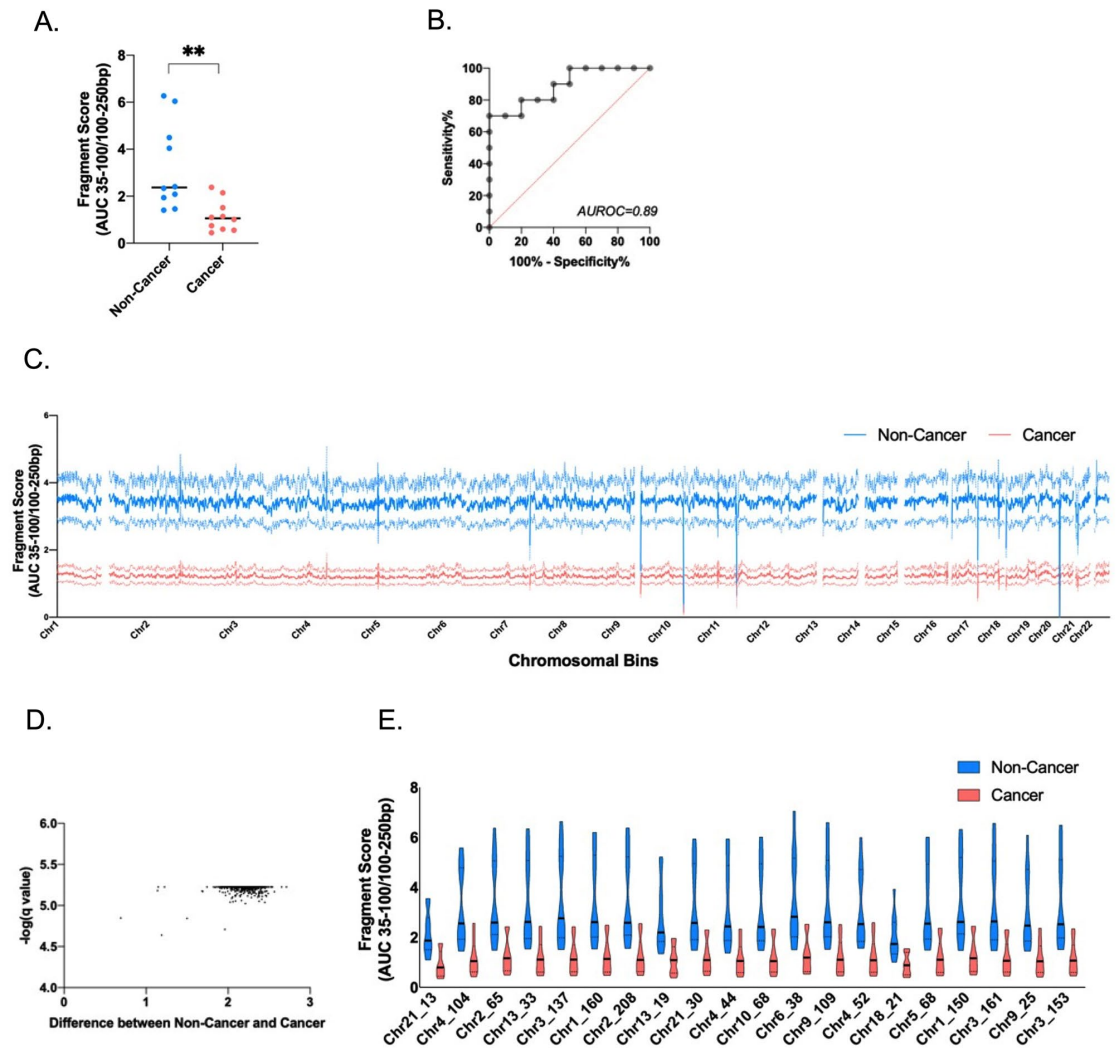
ScfDNA fragmentation pattern and fragmentomics in cancer vs. noncancer donors. (**A**) Fragment score between the cancer and noncancer groups, with each dot representing each sample. p value = 0.0059, Student’s t test, Welch’s correction (**B**) Area under receiver operating curve = 0.89. (**C**) Fragment score for every chromosomal bin for autosomal chromosomes, noncancer donors (Turquoise), and cancer donors (Peach), solid line representing mean, dashed line representing SEM. (**D**) Volcano plot demonstrating over 2700 chromosomal locations with significant differences in fragment scores. Multiple t test, without considering consistency of SD, false discovery rate Benjamini, Krieger and Yekutieli method, p value < 0.05, q value < 0.05. G. Top 20 significant chromosomal locations with different fragment score p values < 0.05, multiple t test, corrected FDR.

As an alternative to the Jagged Peak-Valley index [26], fragmentomic scores representing the relationship between longer and shorter cfDNA fragments are a valuable metric to describe changes in global patterns of cfDNA fragmentation in disease states (Supplementary Fig. 3A). Overall, there was an observable difference, p value = 0.0059, between the ratio of fragments shorter than 100 bp to those longer than 100 bp, where noncancer subjects were more fragmented than GC subjects (Fig. 4A). When individual chromosomal positions were analysed, they revealed a similar pattern: cancer had predominantly longer ScfDNA fragments contributing to a lower fragmentation score when compared to the noncancer group (Fig. 4C). Following analysis of 2887 bins (1 million bps/bin), 2700 chromosomal regions demonstrated a significant difference, with p values ranging from 0.02 to 0.0019 in fragmentomic scores between the two groups (Fig. 4D). The top 20 chromosomal bins demonstrating differences between these two groups were determined (Fig. 4E).

### Functional peak patterns of identity for human ScfDNA

**Fig. 5:**
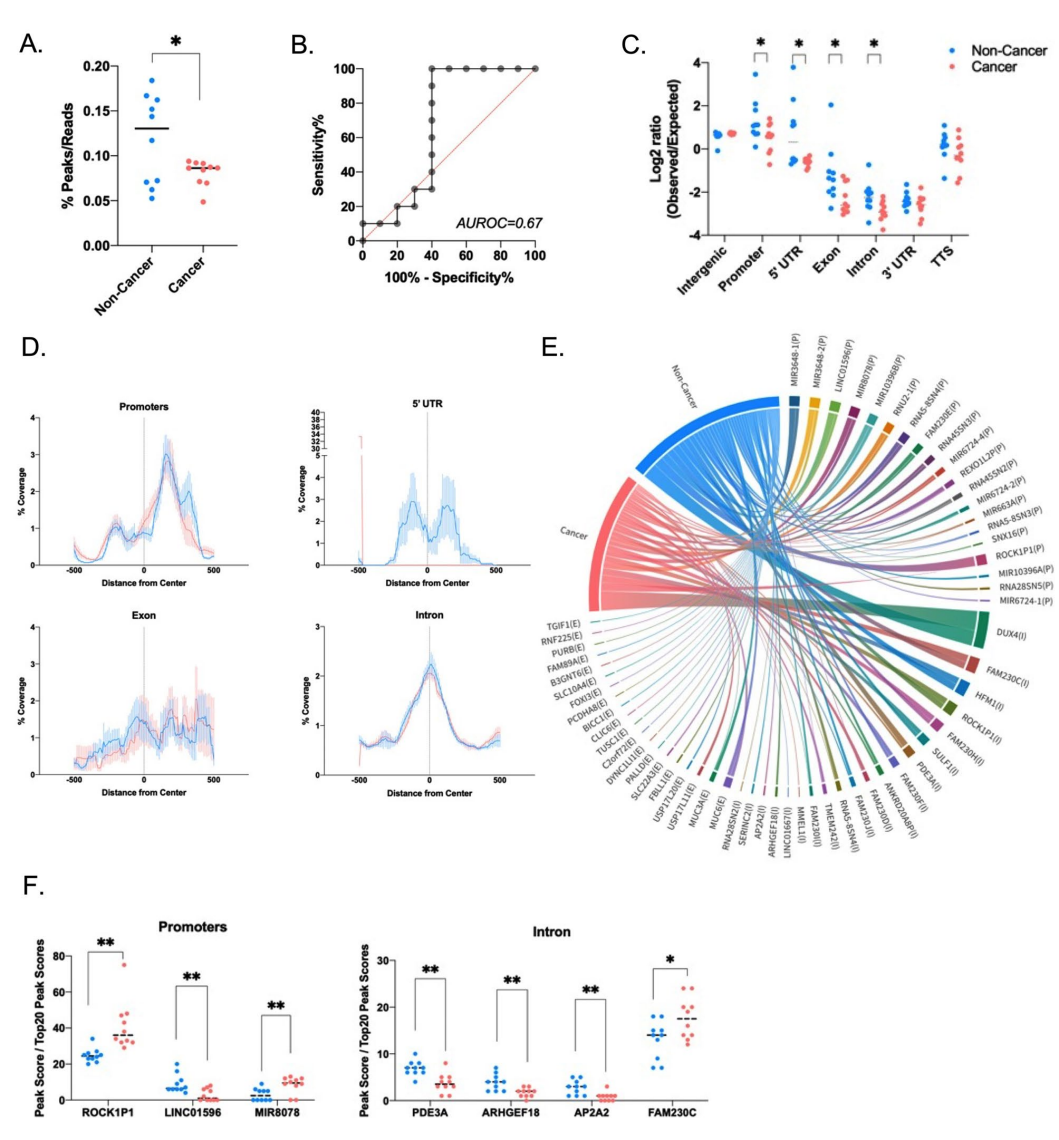
Genetic identity of ScfDNA in cancer vs. noncancer donors. (**A**) Number of peaks for Scf DNA reads, ratio of number of peaks to total number of Scf DNA reads, each dot representing each sample, p value = 0.0447, Student’s t test, Welch’s correction (**B**) Area under receiver operating curve = 0.67. (**C**) Difference between observed over expected peaks formed by ScfDNA in cancer (Peach) in noncancer (Turquoise) cohort, each dot representing each sample, p value < 0.05, Multiple t test without considering consistency of SD, uncorrected. (**D**) The relative coverage of ScfDNA fragments for the 5’ UTR, promoters, introns, and exons from the center of the peak in samples from noncancer (Turquoise) and cancer (Peach) donors. The mean (solid line) and SEM (shading) of the data are shown. (**E**) Chord plot demonstrating different genes contributing to ScfDNA forming significant peaks from promoter, intronic and exonic elements in the cancer and noncancer cohorts. G. Significantly different genes in the cancer and noncancer cohorts contributing to ScfDNA reads from different genomic elements, such as introns and promoters. p value < 0.05, Multiple t test without considering consistency of SD, uncorrected

Since we observed that specific genomic coordinates demonstrated significant chromosomal coverage (Fig. 3E) or fragmentomic (Fig. 4C) differences, we examined whether there were particular sequences of interest. We surveyed the alignment files for regions with naturally converging peaks of ScfDNA reads (Supplementary Fig. 4A). We observed that the total number of peaks formed by the reads in the noncancer cohort was significantly higher than that in the cancer cohort (Fig. 5A&B), with an AUROC of 0.67. Furthermore, the fragments formed peaks in regions associated with different proportions of intergenic, intron, and exon portions. The peaks aligned to the intergenic portions of the genome were higher in cancer, while those mapped to the promoter, intronic, exonic, and 5’UTR portions were higher in the noncancer cohort (Fig. 5C). While evaluating the peaks, we also observed a difference in % coverage of ScfDNA among the various genomic regions for cancer and noncancer cohorts (Fig. 5D, Supplementary Fig. 4B). Promoters and exons demonstrated stark differences in the % coverage downstream from the center of the functional element (Fig. 5D).

Further analysis was performed to establish the genomic identity of the ScfDNA fragment peaks, which identified common genes from different genomic categories (promoter, introns, exons) between the cancer and noncancer cohorts (Fig. 5E). Of these common genes, there were significant differences in a subset of promoter and intron genes (Fig. 5F Supplementary Fig. 4C).

### End-motif features of ScfDNA and G-Quad complexes

**Fig. 6:**
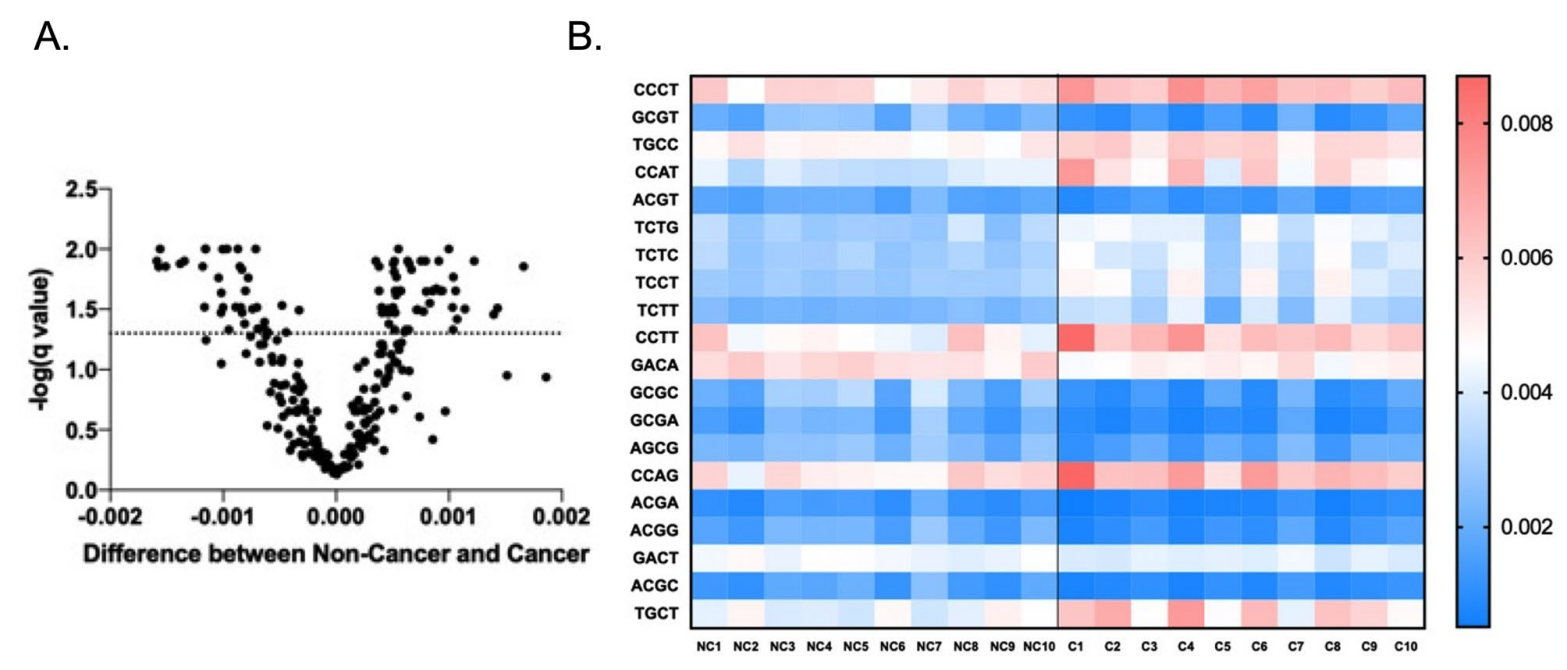
End motif sequences of ScfDNA in cancer vs. noncancer donors. (**A**) Volcano plot demonstrating 4-mer end motifs with significant differences in the ScfDNA reads. Multiple t test, without considering consistency of SD, false discovery rate Benjamini, Krieger and Yekutieli method, p value < 0.05, q-value < 0.05. (**B**) Heatmap demonstrating the top 20 significantly different 4-mer end motifs between cancer vs. noncancer, p value < 0.05, multiple t test, corrected FDR.

Another aspect of fragmentation can be described by the end-motif patterns resulting from specific nuclease activity. The first 4 nucleotides have been described as a valuable metric to differentiate states such as cancer, fetal DNA, or maternal DNA. [19] To estimate the randomness in the occurrence of 4 mer motifs, we calculated Shannon’s entropy and found that cancer had less randomness, as suggested by the reduced Shannon’s entropy (Supplementary Fig. 5A&B). When 256 combinations of possible motifs were considered (Supplementary Fig. 5C), we found 87 significantly different motifs between the two cohorts, with p values ranging from 0.022 − 0.0002 (Fig. 6A&B). A larger proportion of ends in cfDNA in cancer and noncancer donors were mainly guanine-based (Supplementary Fig. 5D).

The G-quadruplex structures in promoter sequences have been identified to play a role in transcriptional regulation. [30] An enrichment of G-quadruplex has been reported to be associated with ss ultrashort cfDNA fragments in plasma. [17, 31] Although not significant in the current data set (Supplementary Fig. 5E&F), we observed that the prevalence of the G-quadruplex sequence in the ScDNA of the noncancer cohort was elevated compared to the GC cohort.

### Microbial origins of ScfDNA

**Fig. 7:**
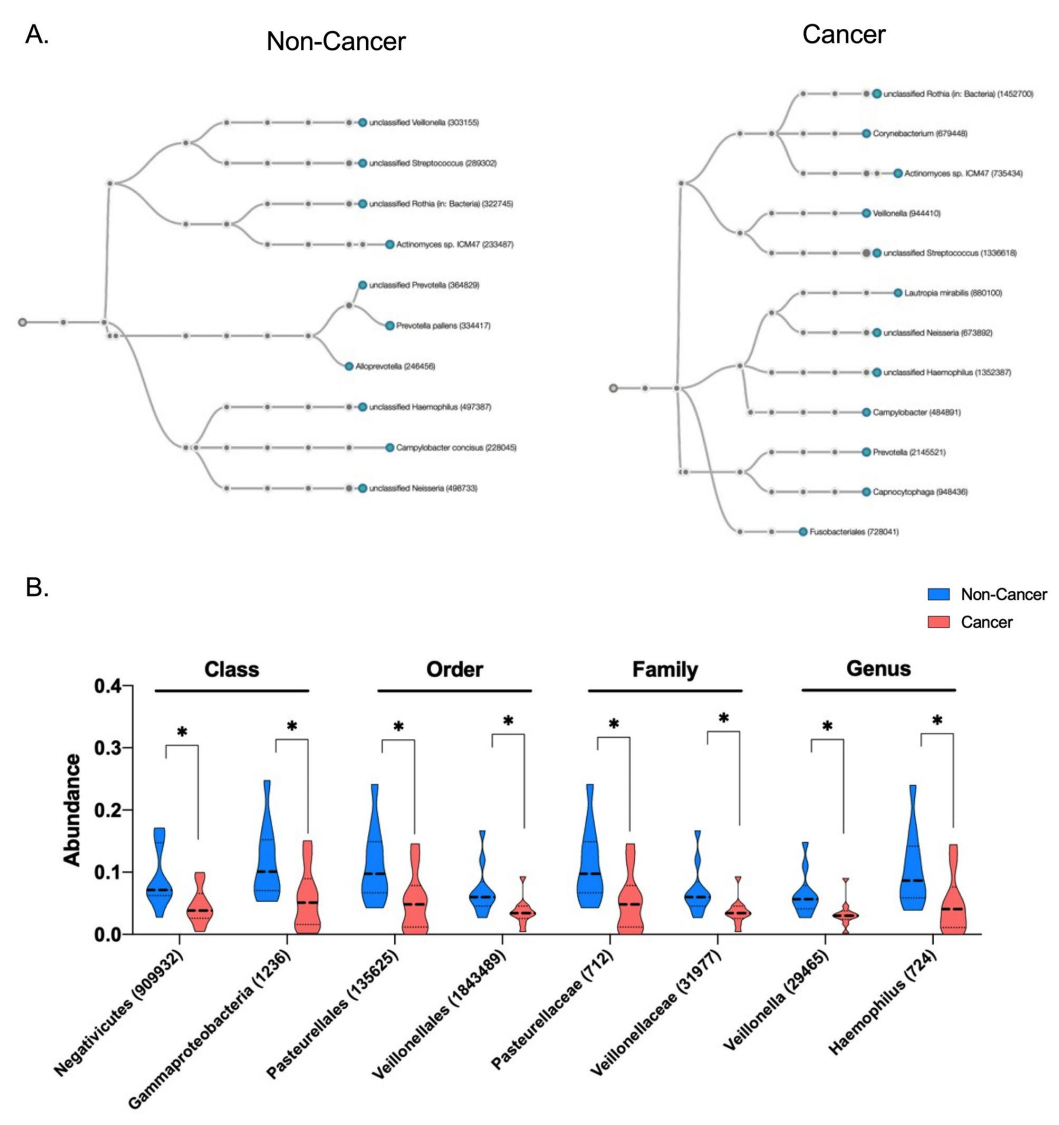
Microbial origins of ScfDNA in cancer vs. noncancer donors. (**A**) Taxonomic chart depicting common microbial species and groups such as Rothia and Streptococcus. (cancer cohort) and Veillonella and Actinomyces (noncancer cohorts) (**B**) Significantly different microbial class, order, family, and genus between the cancer and noncancer cohorts, p value < 0.05, multiple t test, without considering consistency of SD, uncorrected

As part of our sequential alignment strategy, the unmapped sequences were aligned to a microbial reference database after initial alignment to the human reference (hg 38). Aside from the observed increase in microbiota mapping in GC, p value = 0.0361 (Fig. 3D and Supplementary Fig. 6A), we observed a decrease in microbiota diversity in the saliva of GC (Supplementary Fig. 6B). Qualitative differences in the microbial phylogenetic trees were observed. (Fig. 7A, Supplementary Fig. 6C). Specifically, significant differences in the class of microbes contributing to the microbial reads revealed that noncancer subjects had a greater contribution from Negativicutes and Gammaproteobacteria, p value = 0.02 (Fig. 7B). Similarly, the family of microbial species demonstrated a decrease in Pastuerellacae and Veillonelacae, p value = 0.02 and 0.03, respectively, compared with species in cancer from the lactobacilli order. When the microbes were explored at the genus level, a similar trend was observed with Veillonella and Haemophilus, p value = 0.03 and 0.036, respectively (Fig. 7B).

### Integration of biomarkers

**Fig. 8:**
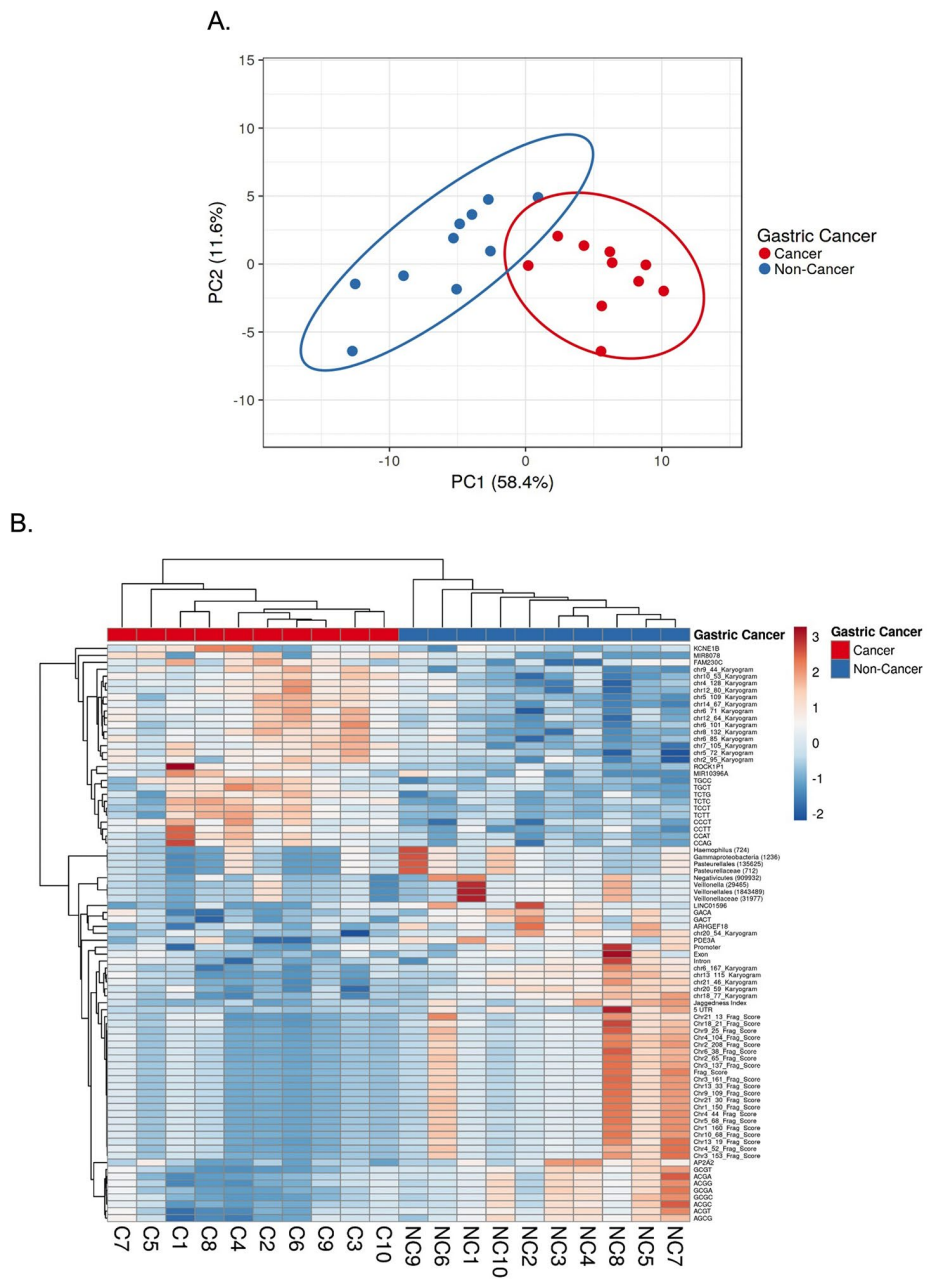
Multivariable analysis of ScfDNA features in cancer vs. noncancer donors. (**A**) Principal component analysis of 92 significant features and targets, demonstrating greater separation in principal component 1. (**B**) Clustering heatmap of significant features and targets in individual cancer and noncancer cohort cases. Rows depict the targets/features of ScfDNA. Columns depict each case in the cancer/noncancer cohort

To integrate the discovered biomarkers, which were significantly different between the two groups (fragmentomics, karyogram, end motif, functional element, microbial population, and mitochondrial bulk), we performed a multivariable analysis using ClustVis. We used dimensional reduction by performing principal component analysis (PCA) [32] (Fig. 8A) and used the top 20 dimensions to identify the most discriminatory features (Supplementary Fig. 7A). The calculated PC1 was most discriminatory for the two groups, with p value < 0.0001 (Supplementary Fig. 7B&C) when compared to PC2 (Supplementary Fig. 7D&E), p value = 0.231, and others (data not shown). In addition to PCA, we also used unsupervised hierarchal clustering, which demonstrated the clustering tree and most differentiating features of the samples of this cohort (Fig. 8B).

## Discussion

Plasma circulating cfDNA has been well studied in its usefulness for prenatal testing, cancer detection, and immune disorders. [33] For cancer LB, mutations in cfDNA have been established as biomarker targets for noninvasive cancer detection and monitoring. However, for many cancers, tumor heterogeneity and lack of specific driver mutations make the detection of mutated ctDNA less useful. The exploration of nonmutation features of cfDNA, which indicate tumor states, is an alternative strategy that can aid in improving the diagnostic abilities of cfDNA. We predict that these features could be assayed alongside conventional mutation detection for overall improvement in liquid biopsy sensitivity.

With increasing interest in alternative biofluids in LB, saliva is attracting sufficient traction to warrant further research. [1] Although protein and RNA have been previously investigated as cancer biomarkers in the supernatant fraction of saliva [4], articulating cfDNA’s useful clinical characteristics is still in its infancy. To this end, we tested the hypothesis that employing BRcfDNA-seq (DNA extraction from the supernatant cell-free component of saliva, ss library preparation, and lcWGS) would identify features of ScfDNA that demonstrated the clinical utility of ScfDNA. Here, we report that aspects of the unique fragmentation pattern and dynamic changes in the microbiome of ScfDNA can be clinically useful as biomarkers for disease states.

We first explored the fragment characteristics of healthy ScfDNA. ScfDNA has a distinctive fragment profile with multiple peaks and a unique jagged pattern size distribution from 20 to 200 bp (Fig. 2). Meanwhile, below 200 bp, plasma cfDNA has been described to consist of two distinct peaks: a mncfDNA peak at 167 bp and a short cfDNA peak at 50 nt [21]. Within these bimodal peaks of plasma cfDNA, mncfDNA DNA is generally ds with jagged ends [26, 34] and nicked ds [35], and the short (~ 50 bp) cfDNA appears to be ss [17, 18, 20, 21, 31]. In contrast to the bimodal peak of plasma cfDNA, ScfDNA lacks the two major peaks, demonstrating a sizable proportion of ScfDNA fragments between 50 and 167 bps. ScfDNA demonstrates a jagged profile, wherein we find similar conformations, ds with jagged ends, nicked ds, ss, and a population of short cell-free DNA (~ 50–70 bp). Moreover, the ScfDNA profile resembled a urinary cell-free DNA profile. However, unlike saliva, the cfDNA of urine has an accentuated jagged pattern from 20 to 200 bp with a more extended modal peak at 80–100 bp. [26, 36].

To demonstrate the clinical utility of ScfDNA, we compared ScfDNA from 10 GC and 10 noncancer donors. We observed that the GC and noncancer ScfDNA size distributions contained a distinct fragment-length silhouette. Further examination showed significant differences in microbial and human (nuclear and mitochondrial) origins and distribution alongside nucleotide characteristics (end motif and G-quad complexes).

Cell-free DNA in plasma or other biofluids has been attributed to cell death or active release, and its apparent structural features are dependent on nuclease activity. [19, 37] It is plausible that the observed features of ScfDNA are influenced by similar mechanisms. We hypothesize that differences in features of ScfDNA highlighted in our report reflect a complex interplay between cell death mechanisms, nucleases, and microbiome activity, which are affected during the disease state.

The fragment profiles of the two cohorts had common fragments below 100 bp; the cancer cohort demonstrated an additional peak at ~ 160 bp, which may represent a greater mononucleosomal DNA contribution (Fig. 3A), as in plasma. The occurrence of cfDNA fragments of lengths mononucleosomal DNA can be attributed to apoptosis-associated nontargeted DNA fragmentation. Apoptosis has been identified as a critical mechanism in GC progression. [38] Moreover, we also observed a significant difference between the ratio of shorter and longer ScfDNA fragments between cancer and noncancer controls, wherein shorter fragments were in greater proportion in the noncancer cohort (Fig. 4C). These findings corroborated the findings comparing ALU fragments in oral cancer and noncancer samples. [39].

Between GC and noncancer tissues, we observed a difference in ScfDNA fragment peaks mapping functional portions such as intron, exon, and promoter changes (Fig.s 6 C & D). Changes in nucleosome positioning [15] or the activity of nuclear protein–DNA interactions are tightly linked to the specific gene expression of each cell type. Therefore, one possible explanation for the apparent profile of ScfDNA peaks from promoter, intron, or exon regions is that they originate from nucleosome-depleted regions (NDRs) [40]. NDR portions are more susceptible to DNA fragmentation by nucleases. [40].

The appearance of released DNA also reflects the activity of various nucleases, yielding variations in fragment length [16] or end motif diversity [19]. Previous reports have shown that the occurrence of C- or T-rich end motif sequences is indicative of cleavage by DNASE1L3 and DNASE1 enzymes. As observed, the C-rich motif had more abundance in normal saliva, and we can speculate a similar enzymatic process, contributing to features of ScfDNA (Fig. 2F). These 4-mer/6-mer end motifs of cfDNA have been shown to be diagnostically helpful due to disruption in the activity of nucleases [34, 41], which is also reflected in our results (Fig. 7B).

Since various cell types in the oral cavity are associated with the expression of specific nucleases, we suspect that the activity of these nucleases may play a significant role in ScfDNA fragment presentation. For example, DNAse2, DNAse1L2, and TREX2 in oral epithelial cells; [42, 43] DNAse1 and DNAse1L3 in salivary glands; [44] and other nucleases such as ‘deoC’ or ‘nuc’ are secreted by oral microbiota. [45, 46] Additionally, the aberrant activity of nucleases such as DNAse1, DFFB, XPF/XPG, etc., has been reported in GC patients. [47, 48] Thus, the perceived variation in ScfDNA fragment profiles in healthy and disease states could be explained by a disruption of nuclease activity within the oral cavity. Additionally, since oral microbiota changes dramatically during gastric and gastroesophageal cancers, [49–51] it is highly plausible that altered nuclease activity associated with oral microbiota may further contribute to the observed features of ScfDNA in diseased subjects. The preliminary data shed some light on the possibility of oral microbiota and DNase1L3 affecting ScfDNA, warranting further exploration.

Another structural feature in plasma cfDNA is the occurrence of G-quadruplex structures. G-quadruplex structures are suggested to be important transcriptional regulators. [30] It has been noted that there is an increased retention of G-quadruplex structures in cancer cells, especially GC cells, [52, 53] while plasma cfDNA demonstrates reduced G-quadruplex structures in cancers [17]. We observed a similar trend of reduction in ScfDNA with reduced G-quadruplex structures.

Interestingly, the current landscape of LB for GC detection relies on plasma-based assays for miRNA detection and ctDNA detection with an AUROC ranging from 0.675–0.88, [54] while the performance of ScfDNA on the pilot cohort for individual to integrated features ranges from 0.65 to 0.99, demonstrating the promising potential of ScfDNA for LB assay development.

It should be noted that while these specific features and analysis strategies for ScfDNA are promising, they were observed only in a small cohort. Thus, further validation in PRoBE-compliant setting and sufficiently powered cohort is required before they can be considered for clinical deployment [55]. Demographic factors such as physiological age, ethnicity, gender and smoking history could contribute to the distinct findings we have reported. To alleviate these concerns, validating these findings on a large cohort with matched controls for demographic factors and PRoBE-compliant settings will allow the biomarkers to be definitively validated in a clinical context of use. For example, with validation, a fragment score below or above 2.0 could potentially be considered an indicator of gastric cancer (Fig. 4). These thresholds could also be set for almost all metrics presented here.

Additionally, there may still be some future value in determining if circulating tumor DNA signals are embedded in the ScfDNA. Through this study, we illustrated the abundance of short DNA with multiple peaks from 50 to 100 bp. Currently, ddPCR primers and NGS target-capture workflows are not optimized for the DNA of such short characteristics. Their short fragment size restricts the possibilities during primer design, which can affect the sensitivity of the assay [56]. For conventional NGS target-capture workflows, the typical capture probe of 120 bp is not designed for short targets requiring creative approaches to improve the theoretical detection ratio [57]. Thus, for this work, in order to avoid making conclusions from unoptimized methods, we only focus on exploring the whole-genome sequencing data.

One strategy that complements our low-coverage sequencing would be to incorporate an assessment of the copy number aberrations within the ScfDNA of these samples. Most copy number aberration cfDNA tools have been tuned to work for plasma or tissue, but none have been designed for ScfDNA [58–60]. We are actively exploring this direction and foresee this as another aspect that can be added to our pre-existing toolbox for ScfDNA analysis.

## Conclusion

Cell-free DNA obtained from saliva demonstrates a jagged pattern, two major peaks with multiple small peaks, and a 10-bp periodicity. To the best of our knowledge, the appearance of a 10-bp periodicity and jagged end pattern of ScfDNA has not previously been reported. Moreover, ScfDNA demonstrates the potential to be a promising biomolecule for GC detection. The jagged profile appearance alone was able to differentiate between GC and noncancer controls. In addition to peak-valley index, ScfDNA fragmentomics, 4-mer DNA end-motif profile, and origins from microbial sequences demonstrate promising clinical utility. As an emerging field of cfDNA-based liquid biopsy, ScfDNA will have many future research and diagnostic applications.

### Electronic supplementary material

Below is the link to the electronic supplementary material.

**Supplementary Fig. 1**. Characteristics of salivary cell-free DNA. (A) Electrophoretic visualization of cell free DNA extracted from supernatant fraction of spun down saliva. A band at ~ 150 bp suggestive of mononucleosomal cell free DNA and dark band above 1500 bp suggestive of genomic DNA. (B) Electrophoretic visualization of ScfDNA treated with Arcticzyme (Ae) (Double Stranded DNAse), Exonuclease (Exo) (Single Stranded Nuclease), PreCr (N) (DNA Nick Repair enzyme), prepared with Single and Double Stranded Library, suggesting occurrence of multiple conformations of ScfDNA. Cell free DNA band visualized at 300 bp and 200 bp, suggestive of ~ 160 bp mononucleosomal cfDNA and ~ 50 bp for ultrashort cfDNA following 160 bp adapters trimming. (C) Chord plot demonstrating different genes contributing ScfDNA forming significant peaks using different library preparation methodologies. **Supplementary Fig. 2**. Differences in fragment lengths and fragmentation patterns between Cancer and Non-Cancer Donors. (A) Jagged peak profile with Peaks (Green Circles) and Valley (Maroon Circles), Peak-valley Frequency (Black dashed line), Interpeak distance (Green dashed line), Intervalley distance (Maroon dashed line). (B) Insert size histogram for mitochondrial reads for non-cancer donors (Turquoise solid line) and cancer donors (Peach solid line) with a single peak at ~ 70 bps. **Supplementary Fig. 3**. Differences in fragment lengths and fragmentation patterns between Cancer and Non-Cancer Donors. A. Fragment score, ratio of fragments ranging from 35 to 100 bp to fragments ranging from 100 to 250 bp. **Supplementary Fig. 4**. Genetic Identity of Scf DNA. (A) Homer peak calling based of cell free DNA reads pile up over a genomic portion. (B) The relative coverage of ScfDNA fragments, for Intergenic, TTS from the center of peak in samples from non-cancer (Turquoise) and cancer (Peach) donors. The mean (Solid line) and SEM (Shade) of the data are shown.C. Top 20 occurring genes, from different genomic element, Intergenic, Promoter, Intron, Exon, Transcription termination site, 5’UTR, 3’UTR between cancer and non-cancer cohort. **Supplementary Fig. 5**. Features of ScfDNA. (A) Shannon score, to demonstrate randomness and diversity of 4-mer motifs between cancer and non-cancer Group, each dot representing each sample., p value = 0.1894, Student t test, Welch’s correction. (B) Area under receiver operating curve = 0.62. (C) Shannon entropy scores for 256 occurring 4-mer motifs for cancer and non-cancer cohort (All labels not shown). (D) Top 20 occurring motifs based of Shannon entropy scores in cancer and non-cancer cohort. (E) Percentage of G-Quad complexes in ScfDNA reads between cancer and non-cancer Group, each dot representing each sample., p value = 0.2809, Student t test, Welch’s correction. (F) Area under receiver operating curve = 0.63. **Supplementary Fig. 6**. Microbial origins of Salivary Cell Free DNA. (A) Difference in frequency of Salivary cell free DNA reads, between cancer and non-cancer cohorts p value = 0.0361, Student t test, Welch’s correction. (B) Alpha diversity of microbial population, Shannon score, p value = 0.1936, Student t test, Welch’s correction. (C) Top 18 occurring microbial phyla, 30 occurring microbial class, order, family, genus, species between cancer and non-cancer cohort. **Supplementary Fig. 7**. Multivariable analysis of ScfDNA features. (A) Individual (solid purple line) and cumulative (solid brown line) variance of cancer and non-cancer donors for various Principal Component Indexes. (B) Principal Component 1 scores demonstrate differences between cancer and non-cancer groups; each dot represents each sample. Statistical significance of p-value < 0.0001, Student t-test, Welch’s correction. (C) Area under receiver operating curve = 0.99. (D) Principal Component 2 scores demonstrate differences between cancer and non-cancer groups; each dot represents each sample. Statistical significance of p-value = 0.231, Student t-test, Welch’s correction. (E) Area under receiver operating curve = 0.68. **Supplementary Table 1**. Clinical Characteristics of Gastric Cancer Patients (10 cases). **Supplementary Table 2**. Clinical Characteristics of Non-Cancer (Gastritis) Patients (10 cases).

## Data Availability

The raw fastq.gz files can be found at https://dataview.ncbi.nlm.nih.gov/object/PRJNA999038.

## List of Abbreviations

cfDNA: Cell-free DNA
ScfDNA: Salivary cell-free DNA
ctDNA: Circulating tumor DNA
GC: Gastric Cancer
BRcfDNA-Seq: Broad-Range Cell free DNA-Seq
ss: Single stranded
ds: Double stranded
SOP: Standard operating procedure

## Code availability

Additional analysis command lines, fragmentomics, end motif detection, and G-quad prevalence can be found in the BRcfDNA-seq Suite at WLab a GitHub, https://github.com/WlabUCLA/BRcfDNA-Seq. Any additional underlying code for this will be made available to qualified researchers on reasonable request from the corresponding author.
