# Correction: Multi-faceted attributes of salivary cell-free DNA as liquid biopsy biomarkers for gastric cancer detection

**DOI:** 10.1186/s40364-023-00542-0

**Published:** 2023-11-09

**Authors:** Neeti Swarup, Jordan Cheng, Irene Choi, You Jeong Heo, Misagh Kordi, Mohammad Aziz, Akanksha Arora, Feng Li, David Chia, Fang Wei, David Elashoff, Liying Zhang, Sung Kim, Yong Kim, David T.W. Wong

**Affiliations:** 1 School of Dentistry, University of California, Los Angeles, 90095 Los Angeles, CA USA; 2 The Samsung Advanced Institute for Health Sciences & Technology (SAIHST), Samsung Medical Center, Sungkyunkwan University School of Medicine, 06355 Seoul, Republic of Korea; 3 Indraprastha Institute of Information Technology (IIIT), Delhi, India; 4 Department of Pathology and Laboratory Medicine, David Geffen School of Medicine, University of California, Los Angeles, 90095 Los Angeles, CA USA; 5 Department of Medicine, Biostatistics and Computational Medicine, University of California Los Angeles, 90095 Los Angeles, CA USA; 6 Department of Surgery, Samsung Medical Center, Sungkyunkwan University School of Medicine, 06355 Seoul, South Korea

**Biomarker Research (2023) 11:90**

10.1186/s40364-023-00524-2

The original article [1] contains errors in the attributions for the following co-authors: David Chia, David Elashoff, Liying Zhang, and Sung Kim.

The corrected affiliations can be viewed in the authorship list of this Correction article.

