# Supplementary material for: Multi-faceted attributes of salivary cell-free DNA as liquid biopsy biomarkers for gastric cancer detection
Source: Biomark Res. 2023 Oct 10;11:90. doi: 10.1186/s40364-023-00524-2 (PMC10566128; doi:10.1186/s40364-023-00524-2)
Supplement: Supplementary file 1 — Supplementary Fig. 1. Characteristics of salivary cell-free DNA. (A) Electrophoretic visualization of cell free DNA extracted from supernatant fraction of spun down saliva. A band at ~ 150 bp suggestive of mononucleosomal cell free DNA and dark band above 1500 bp suggestive of genomic DNA. (B) Electrophoretic visualization of ScfDNA treated with Arcticzyme (Ae) (Double Stranded DNAse), Exonuclease (Exo) (Single Stranded Nuclease), PreCr (N) (DNA Nick Repair enzyme), prepared with Single and Double Stranded Library, suggesting occurrence of multiple conformations of ScfDNA. Cell free DNA band visualized at 300 bp and 200 bp, suggestive of ~ 160 bp mononucleosomal cfDNA and ~ 50 bp for ultrashort cfDNA following 160 bp adapters trimming. (C) Chord plot demonstrating different genes contributing ScfDNA forming significant peaks using different library preparation methodologies. Supplementary Fig. 2. Differences in fragment lengths and fragmentation patterns between Cancer and Non-Cancer Donors. (A) Jagged peak profile with Peaks (Green Circles) and Valley (Maroon Circles), Peak-valley Frequency (Black dashed line), Interpeak distance (Green dashed line), Intervalley distance (Maroon dashed line). (B) Insert size histogram for mitochondrial reads for non-cancer donors (Turquoise solid line) and cancer donors (Peach solid line) with a single peak at ~ 70 bps. Supplementary Fig. 3. Differences in fragment lengths and fragmentation patterns between Cancer and Non-Cancer Donors. A. Fragment score, ratio of fragments ranging from 35 to 100 bp to fragments ranging from 100 to 250 bp. Supplementary Fig. 4. Genetic Identity of Scf DNA. (A) Homer peak calling based of cell free DNA reads pile up over a genomic portion. (B) The relative coverage of ScfDNA fragments, for Intergenic, TTS from the center of peak in samples from non-cancer (Turquoise) and cancer (Peach) donors. The mean (Solid line) and SEM (Shade) of the data are shown.C. Top 20 occurring genes, from different genom [file 40364_2023_524_MOESM1_ESM.pdf]

Supplementary Data

Supplementary Fig. 1. Characteristics of Salivary cell-free DNA.

Supplementary Fig. 2. Differences in fragment lengths and fragmentation patterns between Cancer and Non-Cancer Donors.

Supplementary Fig. 3. Differences in fragment lengths and fragmentation patterns between Cancer and Non-Cancer Donors.

Supplementary Fig. 4. Genetic Identity of Scf DNA.

Supplementary Fig. 5. Features of ScfDNA.

Supplementary Fig. 6. Microbial origins of Salivary Cell Free DNA.

Supplementary Fig. 7. Multivariable analysis of ScfDNA features.

Supplementary Table 1. Clinical Characteristics of Gastric Cancer Patients (10 cases)

Supplementary Table 2. Clinical Characteristics of Non-Cancer (Gastritis) Patients (10 cases)

Supplementary Figure 1: Characteristics of Salivary Cell Free DNA.

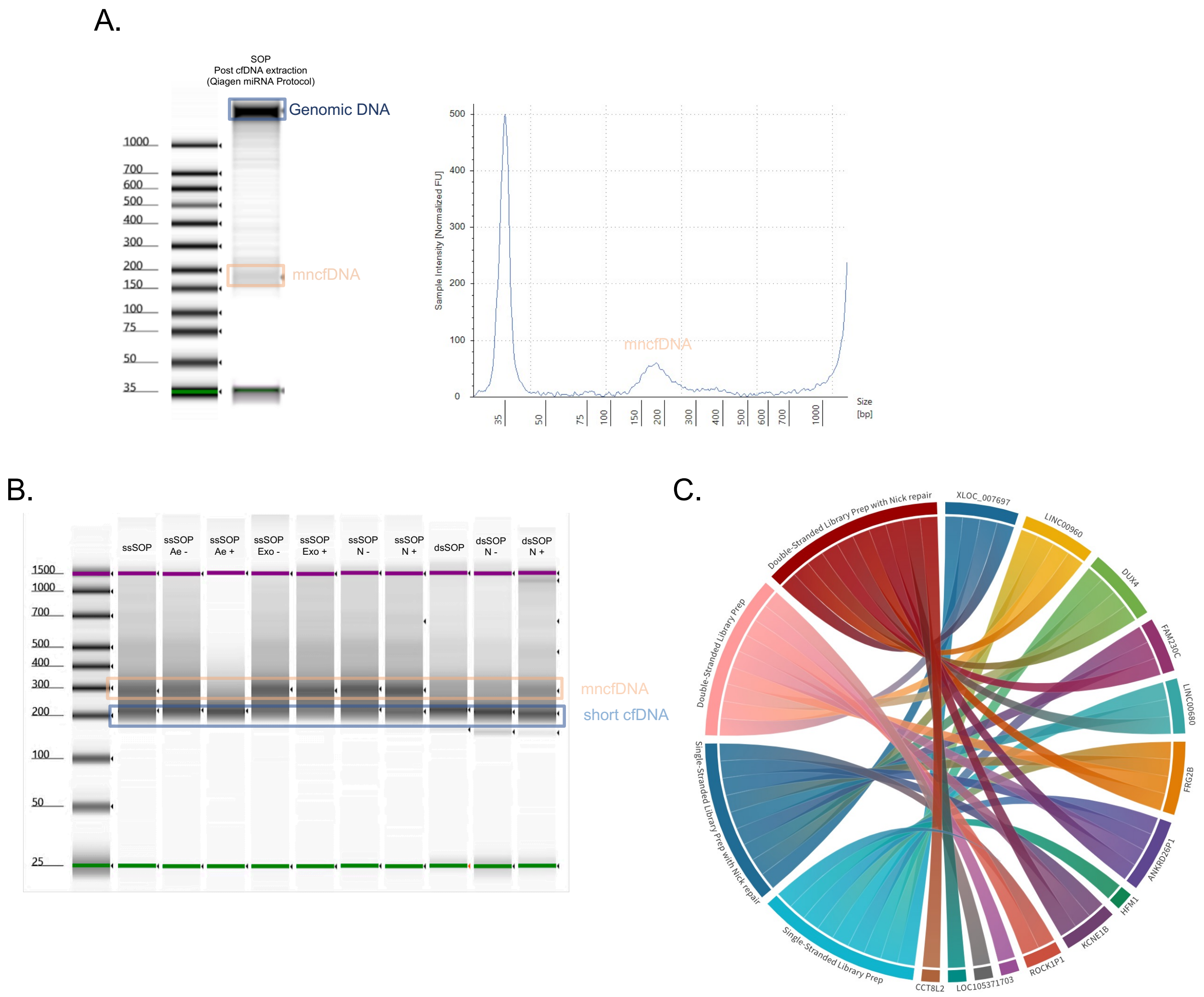

Supplementary Fig. 1. A. Electrophoretic visualization of cell free DNA extracted from supernatant fraction of spun down saliva. A band at ~150 bp suggestive of mononucleosomal cell free DNA and dark band above 1500 bp suggestive of genomic DNA. B. Electrophoretic visualization of ScfDNA treated with **Arcticzyme (Ae)** (Double Stranded DNAse), **Exonuclease (Exo)** (Single Stranded Nuclease), **PreCr (N)** (DNA Nick Repair enzyme), prepared with Single and Double Stranded Library, suggesting occurrence of multiple conformations of ScfDNA. Cell free DNA band visualized at 300bp and 200bp, suggestive of ~160bp mononucleosomal cfDNA and ~50bp for ultrashort cfDNA following 160bp adapters trimming. C. Chord plot demonstrating different genes contributing ScfDNA forming significant peaks using different library preparation methodologies.

Supplementary Figure 2: Differences in fragment lengths and fragmentation patterns between Cancer and Non-Cancer Donors

A.

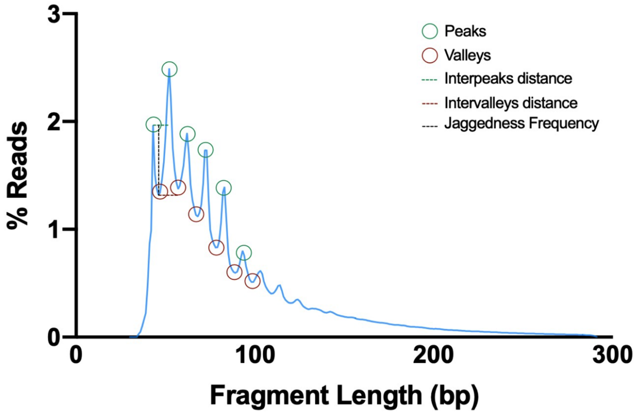

$$Peak - Valley\ Index = \frac{\sum_{i=1}^{n} P_i - V_i}{n}$$

B.

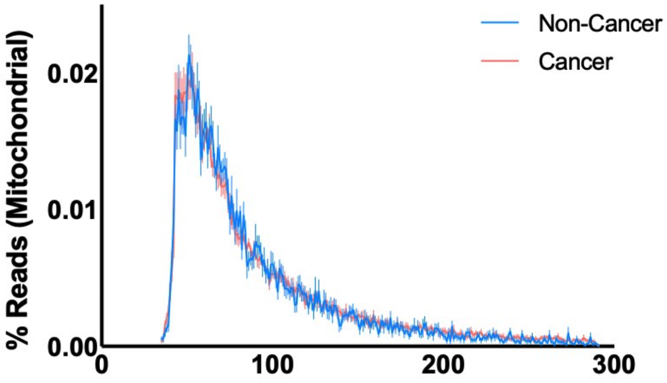

Supplementary Fig. 2. A. Jagged peak profile with Peaks (Green Circles) and Valley (Maroon Circles), Jaggedness Frequency (Black dashed line), Interpeak distance (Green dashed line), Intervalley distance (Maroon dashed line). B. Insert size histogram for mitochondrial reads for non-cancer donors (Turquoise solid line) and cancer donors (Peach solid line) with a single peak at ~ 70 bps.

Supplementary Figure 3: Differences in fragment lengths and fragmentation patterns between Cancer and Non-Cancer Donors

A.

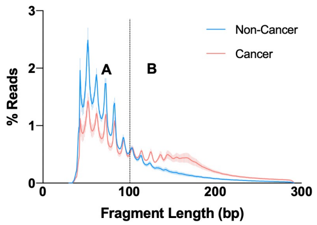

$$Fragment\ Score = \frac{A}{B}$$

Supplementary Fig. 3. A. Fragment score, ratio of fragments ranging from 35-100 bp to fragments ranging from 100-250 bp.

Supplementary Figure 4: Genetic Identity of Scf DNA

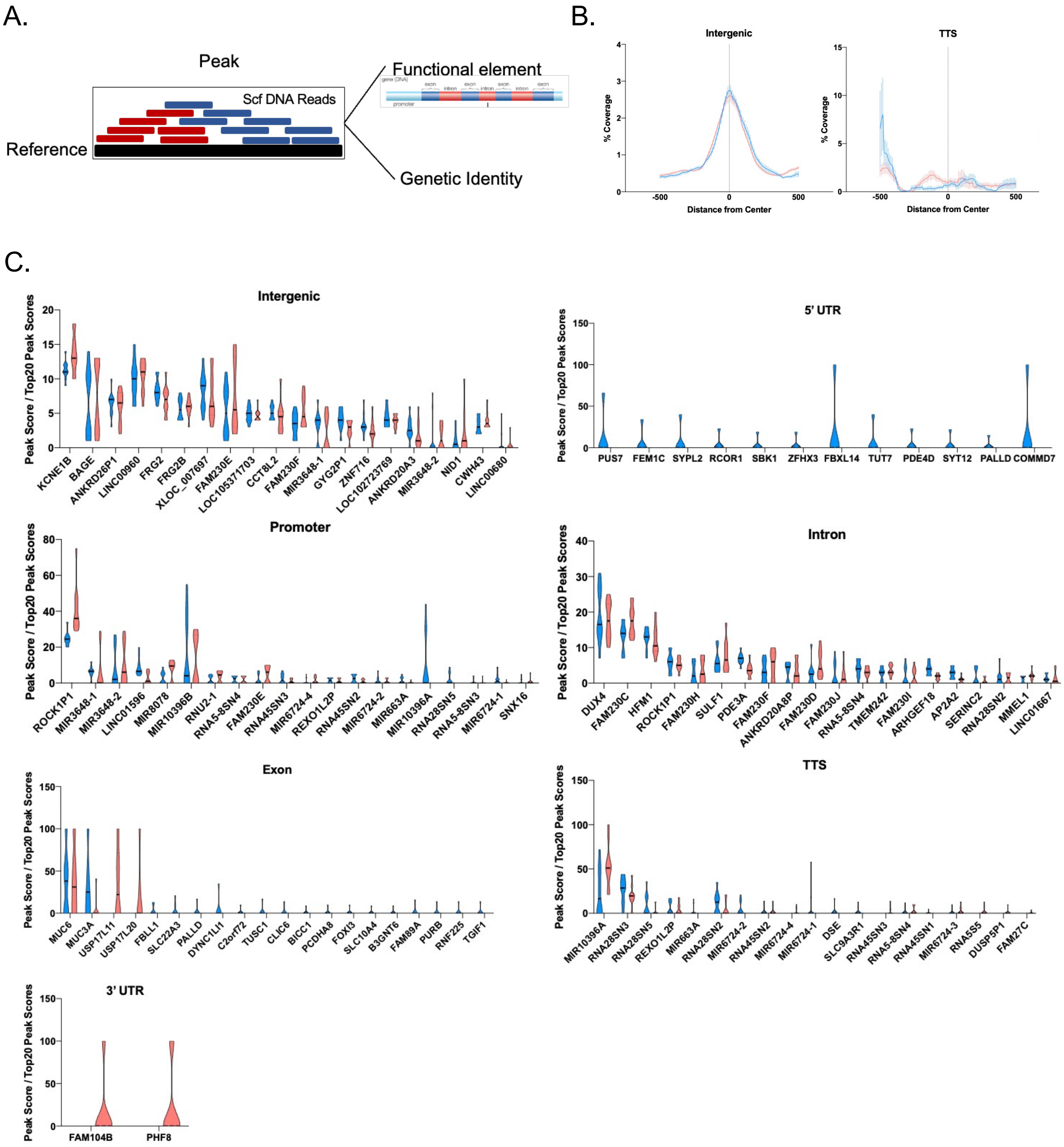

Supplementary Fig. 4. A. Homer peak calling based of cell free DNA reads pile up over a genomic portion. B. The relative coverage of ScfDNA fragments, for Intergenic, TTS from the center of peak in samples from non-cancer (Turquoise) and cancer (Peach) donors. The mean (Solid line) and SEM (Shade) of the data are shown.C. Top 20 occurring genes, from different genomic element, Intergenic, Promoter, Intron, Exon, Transcription termination site, 5'UTR, 3'UTR between cancer and non-cancer cohort.

Supplementary Figure 5: DNA features of ScfDNA

A. B. C.

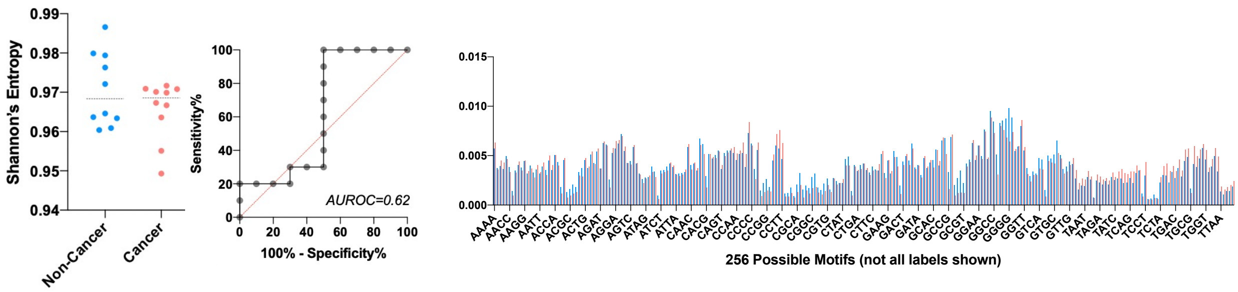

D.

Non-Cancer Top Motif	Cancer Top Motif
GGGG	GGCA
GGCA	CCCA
GGGC	GGTG
GGGT	GGCT
GGCC	GGGT
GGGA	GGGG
GGTG	GGGA
GGCT	CCTG
GGAG	GGCC
CCCA	GGAG

E. F.

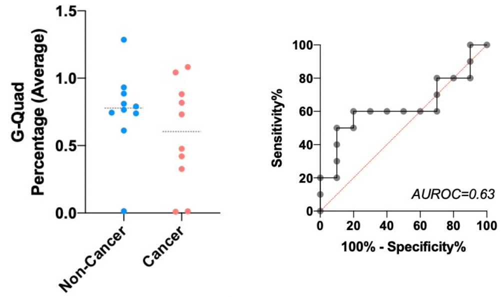

Supplementary Fig. 5. A. Shannon score, to demonstrate randomness and diversity of 4-mer motifs between cancer and non-cancer Group, each dot representing each sample., p value = 0.1894, Student t test, Welch's correction. B. Area under receiver operating curve = 0.62. C. Shannon entropy scores for 256 occurring 4-mer motifs for cancer and non-cancer cohort (All labels not shown). D. Top 20 occurring motifs based of Shannon entropy scores in cancer and non-cancer cohort. E. Percentage of G-Quad complexes in ScfDNA reads between cancer and non-cancer Group, each dot representing each sample., p value = 0.2809, Student t test, Welch's correction. F. Area under receiver operating curve = 0.63

Supplementary Figure 6: Microbial origins of Salivary Cell Free DNA

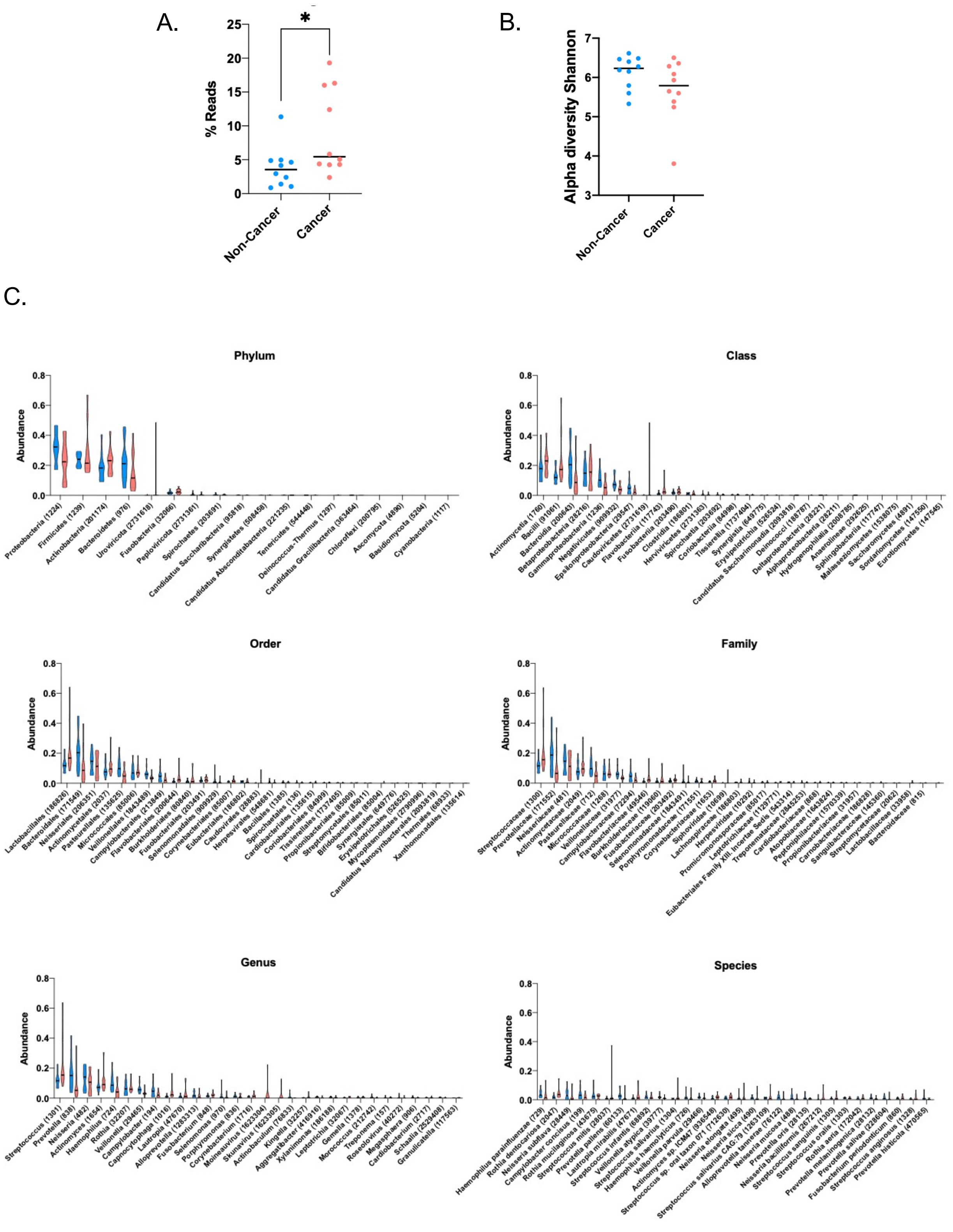

Supplementary Fig. 6. A. Difference in frequency of Salivary cell free DNA reads, between cancer and non-cancer cohorts p value = 0.0361, Student t test, Welch's correction. B. Alpha diversity of microbial population, Shannon score, p value = 0.1936, Student t test, Welch's correction. C. Top 18 occurring microbial phyla, 30 occurring microbial class, order, family, genus, species between cancer and non-cancer cohort.

Supplementary Figure 7: Multivariable analysis of ScfDNA features

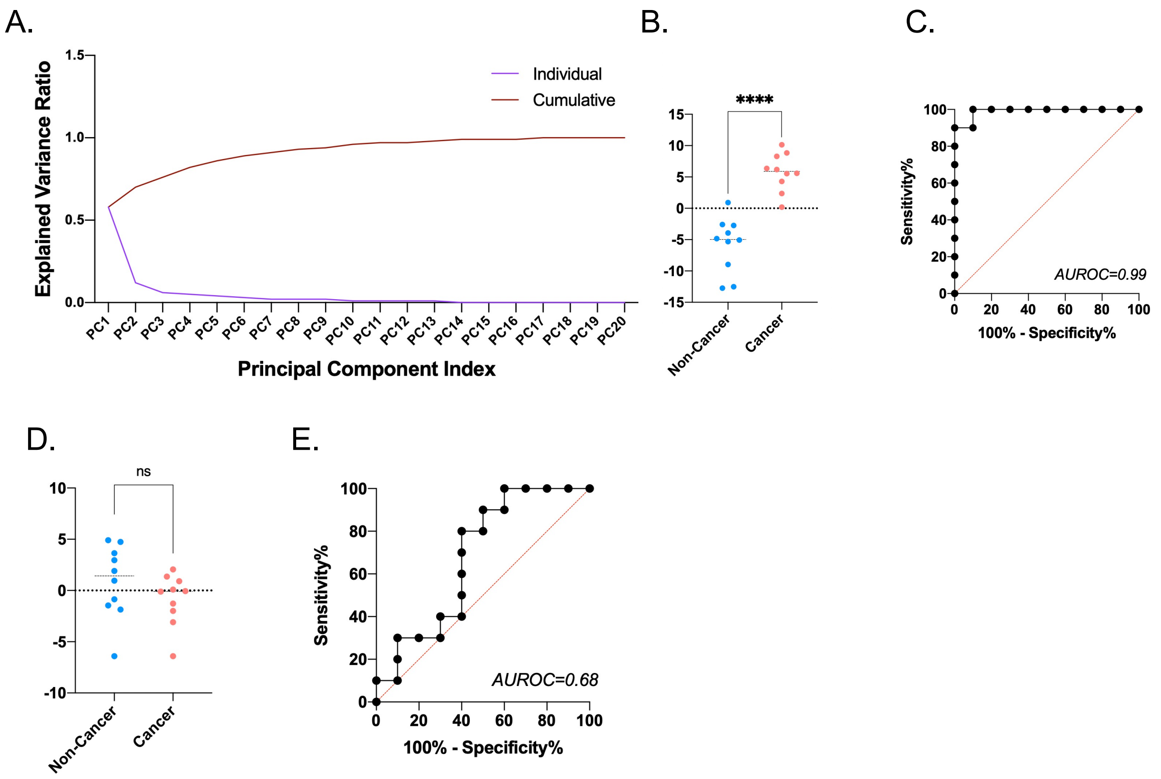

Supplementary Fig. 7. A. Individual (solid purple line) and cumulative (solid brown line) variance of cancer and non-cancer donors for various Principal Component Indexes. B. Principal Component 1 scores demonstrate differences between cancer and non-cancer groups; each dot represents each sample. Statistical significance of p-value < 0.0001, Student t-test, Welch's correction. C. Area under receiver operating curve = 0.99. D. Principal Component 2 scores demonstrate differences between cancer and non-cancer groups; each dot represents each sample. Statistical significance of p-value = 0.231, Student t-test, Welch's correction. E. Area under receiver operating curve = 0.68

Supplementary Table 1. Clinical Characteristics of Gastric Cancer Patients (10 cases)

**Variable**	**No. cases 10 (%)**
Age (y); mean, 59.4	
30-39	1 (10)
40-49	1 (10)
50-59	3 (30)
60-69	3 (30)
70-79	2 (20)
Sex	
Male	5 (50)
Female	5 (50)
T classification	
T1	5 (50)
T4	5 (50)
N classification	
N0	5 (50)
N2/N3	5 (50)
M classification	
M0	9 (90)
M1	1 (10)
Stage	
I	5 (5)
IVa/IVb	5 (5)
H. pylori infection	
Absent	7 (70)
Present	1 (10)
No information	2 (20)

Supplementary Table 2. Clinical Characteristics of Non-Cancer (Gastritis) Patients (10 cases)

Variable	No. cases 10 (%)
Age (y); mean, 40.5	
30-39	4 (40)
40-49	6 (60)
Sex	
Male	5 (50)
Female	5 (50)
H. pylori infection	
Absent	3 (30)
No Information	7 (70)
